# Association of childhood socioeconomic status with edentulism among Chinese in mid-late adulthood

**DOI:** 10.1186/s12903-019-0968-1

**Published:** 2019-12-29

**Authors:** Xiaoning Zhang, Shuang Chen

**Affiliations:** 1 School of Nursing, Xuzhou Medical University, 209 Tongshan Road, Xuzhou, Jiangsu China; 2 The Affiliated Hospital of Xuzhou Medical University, 99 Huaihai West Road, Xuzhou, Jiangsu China

**Keywords:** Socioeconomic status, Tooth loss, Oral health, Edentulism, Mid-late adulthood, Chinese

## Abstract

**Background:**

The aim of this study was to examine the association between childhood socioeconomic status (SES) and edentulism.

**Methods:**

The edentulous status of Chinese in mid-late adulthood was determined using self-reported lost all of teeth from the Health and Retirement Longitudinal Study (CHARLS). Childhood SES was determined based on the following parameters: the education, occupation and working status of the parents; financial situation of the family; relationship with the parents; care, love and affection from the mother; quarrels and fights between parents; primary residence; neighbors’ willingness to help and with close-knit relationships. Adulthood SES was assessed by educational achievements. This study used principal component analysis (PCA) to select variables and binary logistic regression models to determine the association between childhood SES and edentulism.

**Results:**

Data were available from a total of 17,713 respondents, 984 of whom were edentulous (2.9%). The prevalence of edentulism in mid- to late-age Chinese individuals was higher in those with poor childhood SES. In final regression model, edentulism was significantly associated with willingness of neighbors to help with close-knit relationships (*OR* = 0.89, 95% *CI* = 0.79–0.99), parents with high school education or above (*OR* = 1.18, 95% *CI* = 1.01–1.39) and drinking and smoking habits of the father (*OR* = 1.10, 95% *CI* = 0.97–1.24).

**Conclusion:**

Childhood SES was significantly associated with the prevalence of edentulism in mid- to late-age Chinese individuals. In particular, parents with high school education or above, unwillingness of neighbor to help with close-knit relationships, drinking and smoking habits of the father independent of adulthood SES were significantly associated with edentulism. Accordingly, the development of optimal recommendations and more effective intervention strategies requires considering the experiences in early life associated with poor SES contributes to poor oral health.

## Background

According to the World Health Organization (WHO) Study on global AGEing and adult health (SAGE) (2007–2010), the prevalence of edentulism in China is between 8.0–9.0% [1]. Edentulism or toothlessness, the state of having lost all natural teeth, is a worldwide public health issue [2], especially in low- and middle-income countries (LMICs), due to its high prevalence and associated disability [1]. Monitoring edentulism is a determining factor in the assessment of the performance of the oral health care services and adequacy of population health surveillance system [3]. Edentulism is an irreversible condition, which acts as the final marker of oral disease burden and has oral health consequences that include impaired masticatory function, unhealthy diet, and poor oral health quality of life [4]. Edentulism has been associated with coronary heart disease, stroke, and all-cause mortality [5], as well as with a negative impact on life quality, due to pain, infection, speech difficulties and decreased self-esteem [6]. A recent study investigated the potentially deleterious physical and social effects of edentulism and indicated that living with edentulism is associated with depression [7].

The theory of the life course epidemiology proposes that social and economic exposures during certain specific developmental period in life (e.g. childhood and adolescence) [8], have potent and long-term effects on health outcomes in later life [9]. Socioeconomic status (SES) is a major determinant of oral health and prevention of oral diseases [10], and is a high priority for prevention efforts [11]. SES, one of the most important determinants of tooth loss among elders [12], is associated with increased psychological distress, which influences immune function, thereby raising the risk of periodontal diseases. Multiple studies reveal that oral health is more prevalent in poor populations [13], and is associated with SES [14, 15], such as education [16], finance [17], residence [13], social and health behavioral factors [18].

Children with low SES experience greater health problems in adulthood, and aspects of their SES are biologically incorporated through both critical developmental periods and cumulative effects, which relates to poor adulthood health outcomes [19]. Changes of organ systems or physiological processes that occur during critical periods are irreversible, emphasizing that social environments exposures have cumulative effects on health later in life [19]. A dose response effect has been observed between poor childhood SES and adverse health outcomes across different developmental stages of life [20]. Additional studies have indicated that childhood SES exposures are effective predictors of adulthood health outcomes [21]. A direct connection between childhood SES and adulthood health, regardless of whether a child manifests health consequences during childhood or changes SES from childhood to adulthood [19].

The findings from the Survey of Health, Ageing and Retirement in Europe highlighted the long-lasting relationships between childhood living conditions and oral health [22]. Another study based on the Costa Rican Longevity and Healthy Aging Study 1945–1955 Retirement Cohort, indicated that SES in early life has long-term consequences on severe tooth loss [23]. The New Zealand cohort study found a threefold increase in adult periodontal disease and caries in low versus high childhood SES groups [24]. Few studies based on birth cohort analysis have been reported, one study revealed that changes in advantage or disadvantage in childhood are associated with oral caries and tooth loss in adulthood [25]. Studies to date have not focused on childhood SES and later edentulism [26–28].

Family factors play a critical role in shaping the life course of and individual [29], family SES has been validated and used as a classifying variables of childhood SES, which help understand the relationships between childhood SES and oral health, such as occupation of the parents [24], education and work status of the parents [29], quarrels and fights between parents, relationships with parents and financial situation of the family [29]. Previous studies indicate that children whose parents are farmers are more likely to develop edentulism in adulthood [23, 30]. A Korean study determined that education of the parents is associated with the tooth loss status of elders [31]. Like family SES, low levels of conflict, and loving and caring family relationships, the care, love and affection from the mother, also influences family functions [21]. Few studies highlight that health-related behaviors of the father, such as smoking and alcohol consumption contribute to adulthood diseases [32]. Children of parents who have marital conflict may have difficulties in social competence and maintaining close relationships [21].

Childhood SES has been suggested as a predictive factor for oral health [1], and inequality with respect to oral health services [7]. Poor childhood SES affects medical access [33], and lack of access to oral health services may contribute to tooth loss. Thus, the prevalence of severe tooth loss remains high among adults from poor childhood SES groups [34]. A recent review revealed evidence linking neighborhood exposures to high risk of obesity [35]. Barriers to vaccination access among low-SES children should be better understood [36]. A recent study reported that the variables of childhood SES including residential community security [37], and exposure in early life to food avalibility, are linked with the risk of dyslipidemia [38].

## Conceptual framework and hypothesis

**Fig. 1:**
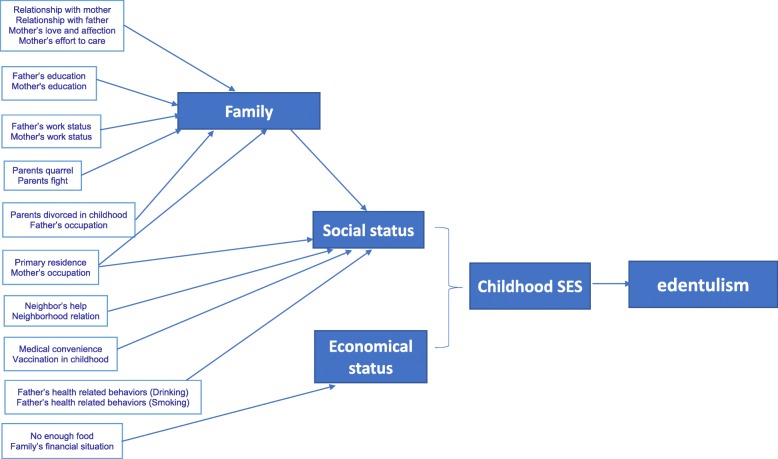
Association of childhood socioeconomic status with edentulism among Chinese in mid-late adulthood

The variabes of childhood SES can be used independently or correlated into a set, Fig. 1 shows the relationships among variables underlying the exposures and outcomes, as well as the data analysis strategy based on the relationships among the variables.

No birth cohort study has been conducted with the data of the Chinese group. To the best of our knowledge, this is the first study to examine the association between childhood SES and edentulism in China. The hypothesis is that childhood SES contributes to edentulism.

## Methods

### The sample

This study analyzed a nationally representative data from the China Health and Retirement Longitudinal Study (CHARLS), using a steady-state design, from 450 villages or communities in 150 counties or districts of 28 provinces. The baseline survey was conducted in 2011, through a four-stage, stratified, cluster probability sampling design [39]. The surveys in 2013, 2014 and 2015 contained the assessments of social, economic and health status, and recruited additional individuals who had just become 45 years old. Further details are provided elsewhere [39], this study combined data from the baseline survey and life history in rounds 2, 3 and 4 surveys. The exclusion criteria were as follows: (i) those individuals who did not report an edentulous status; (ii) age was less than 45 years old; and (iii) those with missing data.

The sample size was calculated based on the prevalence of Chinese edentulism derived from the SAGE (8.0–9.0%) [1], according to the calculation formula below [40], the marginal error was within 2% with 95% confidence level, *P* = 0.5, thus the estimated maximum sample size was 692.

$$ \mathrm{N}=\frac{{{\mathrm{Z}}^2}_{\frac{\alpha }{2}}\kern0.5em P\left(1-P\right)}{d^2} $$

### Measure

Edentulism was assessed based on the response question: “Have you lost all of your teeth?”, the variable had two options (yes or no).

Basic information included sex and age which were categorized into four groups: “45–59”, “60–69”, “70–79”, and “80 or more”.

### Childhood SES

The education of parents consisted of “illiterate”, “elementary school”, “middle school”, and “high school or above”.

The occupation of the parents was categorized into “farmer” and “non-agricultural”.

The parents working status was divided into “all of childhood” and “part of childhood”.

Relationships with parents was based on the question: “How would you rate your relationships with your female/male guardian when you were growing up?” were classified as “excellent”, “very good”, “good”, or “poor”.

Quarrels and fights between parents were classified as “often”, “sometimes”, “not very often”, or “never”.

Parents divorced during childhood was divided into “yes” or “no”.

Care from mother to her child was based on the question: “How much effort did your female guardian put into watching over you?”, which was classified into “a lot”, “some”, “a little”, or “none at all”.

Love and affection from mother to her child was based on the question: “How much love and affection did your female guardian give you while you were growing up?” was classified as “often”, “sometimes”, “rarely”, or “never”.

Father’s health-related behaviors (drinking/smoking) were divided into “yes” or “no”.

Neighbors’ help was based on the question: “Were the neighbors of the place where you lived as a child willing to help each other out?” included “willing to”, “somewhat willing to”, or “unwilling to”.

Neighborhood relationship was based on the question: “Were the neighbors of the place where you lived as a child very close-knit?” included “very close-knit”, “somewhat close-knit”, or “not close-knit”.

Financial situation of the family was based on the question: “When you were a child under 17 years old, compared to the average family in the same community/village at that time, how was the financial situation of your family?”, which was classified into “a lot better off than them”, “somewhat better off than them”, “same as them”, “somewhat worse off than them”, “a lot worse off than them”.

Food availability was based on the question: “When you were a child before under 17 years old was there ever a time when your family did not have enough food to eat?” included “Yes” or “No”.

Primary residence was divided into “rural” or “urban”.

Medical convenience was based on the question: “Are you satisfied with the quality, cost, and convenience of local health care” included “yes” or “no”.

Vaccination in childhood was based on the question: “Before you were 15 years old (including 15 years old), did you receive any vaccinations?” included “yes” or “no”.

### Adulthood SES

Adulthood SES was assessed by adult educational achievements [41], divided into four groups (illiterate, elementary school, middle school, or high school and above).

### Statistical analysis

The differences in edentulism by age, sex, childhood SES and adulthood SES were analyzed using the Chi-Square tests. This study used principal component analysis (PCA) to determine the main factors of childhood SES. The participants with missing data on variables of interest were excluded. In order to evaluate the representativeness of the sample, the age and sex were compared between the excluded and included participants [42]. There was no statistically significant difference between these two groups.

Binary logistic regression was used to examine the associations between childhood SES and edentulism. Age and sex were adjusted in model 1; adulthood SES was subsequently adjusted in model 2. The odds ratio (*OR*) and the 95% confidence interval (*CI*) are presented. The Hosmer-Lemeshow goodness-of-fit test was used to evaluate the multivariable model fit. The statistical significance was considered as two-sided *P* < 0.05. All analyses were conducted using the Stata 14 software (Stata Corp. LLC, College Station, Texas, USA).

## Results

### Sample characteristics

The descriptive statistics and univariate analysis are shown in Table 1. Edentulous status was reported for 17,713 respondents (8498 males and 9215 females), with mean age of 62.9 ± 10.5 years, age range of 45–108 years. A total of 984 respondents were edentulous (2.9%). Individuals whose father/mother’s occupation was farmer were more likely to be edentulous than those whose father/mother’s occupation was non-agricultural (*P* = 0.015; *P* = 0.005). Individuals whose father/mother’s education was high school or above were more likely to be edentulous than those whose father/mother’s occupation was high school and above (*P* < 0.001). Individuals whose parents often quarrel/fight were more likely to be edentulous than those whose parents quarrel/fight less often (*P* < 0.001; *P* = 0.022). Individuals whose neighbors were unwilling to help were more likely to be edentulous than those whose neighbors were more willing to help (*P*<0.001). Individuals who had poor relationships with their mother were more likely to be edentulous than those who had excellent relationships with their mother (*P* = 0.015). Additionally, individuals who had no close-knit neighborhood relationships were more likely to be edentulous than those who had close-knit neighborhood relationships (*P* = 0.001). Also, individuals whose primary residence was in a village were more likely to be edentulous than those whose primary residence was in a city/town during childhood (*P* = 0.005). Individuals who had not enough food were more likely to be edentulous than those who had enough food during childhood (*P* < 0.001). Individuals whose father used to smoke were more likely to be edentulous than those whose father was not a smoker (*P* < 0.001). Individuals who were unsatisfied with medical convenience and did not receive vaccination were more likely to be edentulous than those who were satisfied with medical convenience and received vaccination in childhood (*P* < 0.001). Individuals whose adulthood SES (educational achievements) was illiterate were more likely to be edentulous than those with other educational levels (*P* < 0.001).

**Table 1:** The perveance of main variables among edentulism and non- edentulism

Variables	Edentulism		N (%)	*P*
	YES (%)	NO (%)		
Age				
45–59	137 (1.9)	7124 (98.1)	7261 (41.8)	*<0.001*
60–69	331 (5.6)	5545 (94.4)	5876 (33.2)	
70–79	304 (9.3)	2973 (90.7)	3277 (18.5)	
>80	202 (15.6)	1097 (84.4)	1299 (7.3)	
Gender				
Male	459 (5.4)	8039 (94.6)	8498 (48.0)	*0.598*
Female	515 (5.6)	8700 (94.4)	9215 (52.0)	
Relationship with mother				
Excellent	308 (5.4)	5421 (94.6)	5729 (33.8)	0.015
Very good	259 (4.8)	5133 (95.2)	5392 (31.8)	
Good	165 (5.7)	2730 (94.3)	2895 (17.1)	
Fair	151 (5.4)	2620 (94.6)	2771 (16.4)	
Poor	16 (10.7)	134 (89.3)	150 (0.9)	
Relationship with father				
Excellent	258 (5.2)	4660 (94.8)	4918 (29.9)	0.215
Very good	249 (4.7)	5038 (95.3)	5287 (32.1)	
Good	170 (5.7)	2810 (94.3)	2980 (18.1)	
Fair	168 (5.4)	2920 (94.6)	3088 (18.7)	
Poor	14 (7.0)	186 (93.0)	200 (1.2)	
Mother’s love and affection				
Often	550 (5.4)	9687 (94.6)	10,237 (61.0)	0.474
Sometimes	141 (4.7)	2843 (95.3)	2984 (17.8)	
Rarely	109 (5.5)	1880 (94.5)	1989 (11.9)	
Never	87 (5.6)	1472 (94.4)	1559 (9.3)	
Mother’s effort to care				
A lot	502 (5.3)	8896 (94.7)	9398 (55.9)	0.899
Some	187 (5.2)	3378 (94.8)	3565 (21.2)	
A little	140 (5.1)	2592 (94.9)	2732 (16.3)	
Not at all	63 (5.7)	1040 (94.3)	1103 (6.6)	
Neighbor’s help				
Willing to	419 (5.1)	7815 (94.9)	8234 (47.8)	*<0.001*
Not Willing to	357 (5.1)	6654 (94.9)	7011 (40.7)	
Not very willing to	96 (7.5)	1184 (92.5)	1280 (7.4)	
Not willing to at all	57 (7.9)	661 (92.1)	718 (4.2)	
Neighborhood relation				
Very Close-knit	373 (4.8)	7364 (95.2)	7737 (44.6)	0.001
Somewhat Close-knit	502 (5.7)	8328 (94.3)	8830 (50.9)	
Not Very Close-knit	43 (7.2)	557 (92.8)	600 (3.5)	
Not close-knit at all	19 (10.4)	163 (89.6)	182 (1.0)	
Primary residence				
Village	834 (6.1)	12,923 (93.9)	13,757 (91.1)	0.005
City/town	56 (4.2)	1284 (95.8)	1340 (8.9)	
Mother’s occupation				
Farming	808 (5.5)	13,864 (94.5)	14,672 (93.2)	0.003
Non-agriculture	37 (3.5)	1027 (96.5)	1064 (6.8)	
Father’s education				
No formal education (illiterate)	590 (6.5)	8521 (93.5)	9111 (55.8)	*<0.001*
Elementary school	245 (4.3)	5439 (95.7)	5684 (34.8)	
Middle school	32 (3.6)	853 (96.4)	885 (5.4)	
High school and above	23 (3.6)	613 (96.4)	636 (3.9)	
Mother’s education				
No formal education (illiterate)	867 (6.0)	13,683 (94.0)	14,550 (86.0)	*<0.001*
Elementary school	49 (5.3)	1926 (97.5)	1975 (11.7)	
Middle school	4 (1.6)	247 (98.4)	251 (1.5)	
High school and above	3 (2.1)	141 (97.9)	144 (0.9)	
No enough food				
Yes	702 (5.9)	11,120 (94.1)	11,822 (67.3)	*<0.001*
No	262 (4.6)	5483 (95.4)	5745 (32.7)	
Family’s financial situation				
A lot better off than them	14 (6.9)	188 (93.1)	202 (1.1)	0.168
Same as them	72 (4.8)	1419 (95.2)	1491 (8.5)	
Somewhat worse off than them	480 (5.3)	8504 (94.7)	8984 (51.0)	
somewhat worse off than them	146 (5.3)	2628 (94.7)	2774 (15.8)	
a lot worse off than them	248 (6.0)	3910 (94.0)	4158 (23.6)	
Parents quarrel				
Often	45 (5.2)	826 (94.8)	871 (5.5)	*<0.001*
Sometimes	116 (4.1)	2717 (95.9)	2833 (17.9)	
Not very often	226 (4.4)	4855 (95.6)	5081 (32.0)	
Never	430 (6.1)	6652 (93.9)	7082 (44.6)	
Parents fight				
Often	20 (6.9)	268 (93.1)	288(1.8)	0.022
Sometimes	38 (3.6)	1018 (96.4)	1056 (6.7)	
Not very often	92 (4.5)	1939 (95.5)	2031 (12.9)	
Never	657 (5.3)	11,171 (94.7)	12,428 (78.6)	
Father’s work status				
All of Childhood	861 (5.3)	15,433 (94.7)	16,294 (96.5)	0.378
Part of Childhood	27 (6.1)	415 (93.9)	442 (2.6)	
None of Childhood	9 (6.3)	135 (93.8)	144 (0.9)	
Mother’s work status				
All of Childhood	788 (5.3)	13,967 (94.7)	14,755 (85.6)	0.455
Part of Childhood	54 (6.2)	820 (93.8)	874 (5.1)	
None of Childhood	90 (5.6)	1512 (94.4)	1602 (9.3)	
Father’s health related behaviors (Drinking)				
Yes	74 (6.1)	1136 (93.9)	1210 (6.8)	0.328
No	900 (5.5)	15,603 (94.5)	16,503 (93.2)	
Father’s health related behaviors (Smoking)				
Yes	408 (4.7)	8191 (95.3)	8599 (48.5)	*<0.001*
No	566 (6.2)	8548 (93.8)	9114 (51.5)	
Parents divorced in childhood				
Yes	0 (0.0)	12 (100.0)	12 (0.1)	0.246
No	952 (5.5)	16,493 (94.5)	17,445 (99.9)	
Medical convenience				
Yes	607 (4.7)	12,379 (95.3)	12,986 (91.0)	*<0.001*
No	92 (7.1)	1197 (92.9)	1289 (9.0)	
Vaccination in childhood				
Yes	551 (4.5)	11,755 (95.5)	12,306 (86.6)	*<0.001*
No	143 (7.5)	1762 (92.5)	1905 (13.4)	
Father’s occupation				
Farming	617 (5.5)	10,624 (94.5)	11,241 (82.2)	0.015
Non-agriculture	105 (4.3)	2334 (95.7)	2439 (17.8)	
Adult SES (education attainments)				
No formal education (illiterate)	312 (8.1)	3553 (91.9)	3865 (25.6)	*<0.001*
Elementary school	368 (5.8)	5959 (94.2)	6327 (41.9)	
Middle school	125 (3.9)	3056 (96.1)	3181 (21.1)	
High school and above	44 (2.5)	1687 (97.5)	1731 (11.5)	

### Associations between childhood SES and edentulism

According to the data presented in Table 2, there were 22 possibly correlated variables of childhood SES. PCA was applied to a set of values of linearly uncorrelated variables to synthesize numerous indexes and perform the reduction of variables. Ten main factors were imputed instead of 22 variables (Table 2), the impute factor scores for 62.3% of this sample.

**Table 2:** Component matrix of PCA

Variables					Component						
		1	2	3	4	5	6	7	8	9	10
1	Relationship with mother	0.778									
	Relationship with father	0.705									
	Mother’s love and affection	0.702									
	Mother’s effort to care	0.673									
2	Parents quarrel		0.813								
	Parents fight		0.778								
3	Neighbor’s help			0.818							
	Neighborhood relation			0.803							
4	Primary residence				0.878						
	Mother’s occupation				0.873						
5	Father’s education					0.655					
	Mother’s education					0.619					
6	No enough food						0.710				
	Family’s financial situation						−0.656				
7	Father’s work status							0.750			
	Mother’s work status							0.722			
8	Father’s health related behaviors (Drinking)								0.726		
	Father’s health related behaviors (Smoking)								0.651		
9	Medical convenience									0.671	
	Vaccination in childhood									0.699	
10	Parents divorced in childhood										0.656
	Father’s occupation										−0.739

PCA, principal component analysis

Binary logistic regression analysis was used to examine the associations between these ten factors and edentulism, and the results of these analysis are presented in Table 3. As shown in model 1, sex and age were adjusted, willingness of neighbors to help with close-knit relationships (*OR* = 0.87, 95% *CI* = 0.78–0.96), parents with high school education or above (*OR* = 1.20, 95% *CI* = 1.03–1.39), father used to drink and smoke (*OR* = 1.11, 95% *CI* = 0.99–1.25) were significantly associated with edentulism, and adequate calibration was assessed by the goodness-of-fit test (Hosmer-Lemeshow: χ^2^ = 12.112, *P* = 0.146). As shown in model 2, adulthood SES (educational achievements) was adjusted, willingness of neighbors to help with close-knit relationships (*OR* = 0.89, 95% *CI* = 0.79–0.99), parents with high school education or above (*OR* = 1.18, 95% *CI* = 1.01–1.39), father used to drink and smoke (*OR* = 1.10, 95% *CI* = 0.97–1.24) were significantly associated with edentulism, and adequate calibration was assessed by the Hosmer-Lemeshow test (χ^2^ = 10.149, *P* = 0.237).

**Table 3:** Results of binary logistic regression analysis of the association of childhood SES with edentulism

		Model 1						Model 2					
	Variables	B	S.E.	Wald	df	*P*	OR (95%*Cl*)	B	S.E.	Wald	df	*P*	OR (95%*Cl*)
	Age	− 0.63	0.07	93.06	1	<0.001	0.53 (0.47–0.61)	−0.58	0.07	71.21	1	<0.001	0.56 (0.49–0.64)
	Gender	0.07	0.12	0.31	1	0.58	1.07 (0.85–1.34)	−0.02	0.13	0.03	1	0.86	0.98 (0.76–1.26)
1	Relationship with mother	−0.11	0.06	3.50	1	0.06	0.90 (0.81–1.01)	− 0.11	0.06	3.50	1	0.06	0.90 (0.80–1.01)
	Relationship with father												
	Mother’s love and affection												
	Mother’s effort to care												
2	Parents quarrel	−0.03	0.06	0.26	1	0.61	0.97 (0.87–1.09)	−0.03	0.06	0.29	1	0.59	0.97 (0.86–1.10)
	Parents fight												
3	Neighbor’s help	−0.14	0.05	7.24	1	0.007	0.87 (0.78–0.96)	−0.13	0.06	4.76	1	0.03	0.89 (0.79–0.99)
	Neighborhood relation												
4	Primary residence	0.12	0.08	2.62	1	0.11	1.13 (0.98–1.31)	0.72	0.08	0.84	1	0.36	1.07 (0.92–1.25)
	Mother’s occupation												
5	Father’s education	0.18	0.08	5.41	1	0.02	1.20 (1.03–1.39)	0.17	0.08	4.19	1	0.04	1.18 (1.01–1.39)
	Mother’s education												
6	No enough food	0.07	0.06	1.47	1	0.23	1.08 (0.96–1.21)	0.04	0.06	0.39	1	0.53	1.04 (0.92–1.18)
	Family’s financial situation												
7	Father’s work status	0.09	0.06	0.02	1	0.88	1.01 (0.90–1.13)	−0.01	0.06	0.01	1	0.91	0.99 (0.89–1.11)
	Mother’s work status												
8	Father’s health related behaviors (Drinking)	−0.10	0.06	3.15	1	0.08	1.11 (0.99–1.25)	0.94	0.06	2.35	1	0.01	1.10 (0.97–1.24)
	Father’s health related behaviors (Smoking)												
9	Medical convenience	−0.03	0.05	0.35	1	0.56	0.97 (0.87–1.08)	− 0.02	0.06	0.07	1	0.79	0.99 (0.88–1.10)
	Vaccination in childhood												
10	Parents divorced in childhood	−0.12	0.08	1.99	1	0.16	0.89 (0.75–1.05)	−0.09	0.09	1.07	1	0.30	0.92 (0.77–1.08)
	Father’s occupation												
	Adult SES (education attainment)							0.17	0.08	4.34	1	0.04	1.18 (1.01–1.40)
	Constant	5.12	0.17	882.99	1	<0.001	166.82	4.74	0.26	333.43	1	<0.001	114.81

Model 1: adjusted for age and gender. Model 2: adjusted for age, gender and adult SES. OR, odds ratio; CI, confidence interval; SES, socioeconomic status

## Discussion

To the best of our knowledge, this is the first study to examine the association between childhood SES and edentulism across the life course of mid- to late-age Chinese individuals. Childhood SES was significantly associated with edentulism, parents with high school education or above, unwillingness of neighbors to help with close-knit relationships, and father used to drink and smoke independent of adulthood SES in the regression models remained significantly associated with edentulism in mid- to late-age Chinese individuals. These results supported the conceptual framework and hypothesis that poor childhood SES was significantly associated with edentulism.

The results of this study are consistent with those from previous life-course epidemiological studies on other adulthood diseases [43, 44]. Many studies focused on the economic [45] or environmental [46], factors of the neighborhood in this study highlighted the social perception, willingness of neighbors to help with close-knit relationships independent of adulthood SES remained significantly associated with edentulism in mid- to late-age Chinese individuals. Poor family SES negatively correlated with high quality and affordability of oral health services, and thus those children had restricted access to oral health services. Social support is a positive factor for oral health-related quality of life [19], which is consistent with the results of this study, indicating that children who lived in an environment where neighbors were willing to help with close-knit relationships, were less likely to be edentulous in adulthood. The social characteristics of the neighborhood may influence the psychological development of children. For instance, living with negative neighborhood relationships is associated with poor emotional development, maladaptive social environment and control of feelings [21]. Social inequalities between neighborhoods are related to individual developmental health in early childhood [47]. In addition, living in neighborhoods with low SES is associated with impeding psychosocial support and respect, and contribute to increase stress and feelings of helplessness and isolation, which in turn is detrimental to adulthood health [21].

This study identified an unexpected association between parents with high school education or above and edentulism, suggesting that having parents who had high education did not contribute to decrease the prevalence of edentulism, which is inconsistent with previous studies [48]. The age of respondents was 45+ years old, born between 1950s and 1970s, they went through their whole childhood and early adulthood before1978, when China was mainly an agricultural society and industrially undeveloped. People lacked oral health knowledge during that period of poverty in China, and sugar products were expensive and limited before the reform and opening-up policy; parents who obtained high education were more likely relatively wealthy and could afford to feed their children sugar products. The CHARLS does not contain data about sugar intake during childhood. However, even among the respondents who consumed low amount of sugar, compared to that of sugar-free individuals, the prevalence of edentulism may still be high. Exposure to smoking and drinking environment during childhood has been associated with low educational achievements, and long-term effects on cognitive abilities, which in turn can potentially trigger health damaging behaviors over the life course [21]. Children expose to parental smoking are at increased risk of increased C-reactive protein in adulthood, which may contribute to long-term effects on low-grade inflammation [49]. Regular alcohol consumption by the father is related to low family SES, which predicts less-skilled parenting practices and children’s developmental delay in children [50].

Children who mainly lived with low SES were more likely to have access to inadequate oral health services, which did not meet their basic oral health demands [51]. Medical convenience in childhood was the embodiment of the accessibility to oral health services, which further affected childhood diet quality, oral health and health-related behaviors [52], and may place individuals at higher risk of edentulism [19]. In some LMICs, tooth extraction is the only treatment available for oral problems, which is an important factor relate to edentulism. Inequitable distribution of oral health services may be another factor involved in the association between childhood SES and edentulism, children who did not receive adequate and advanced oral health services and preventive oral health information, that may become vulnerable to oral diseases, which could be avoided with adequate oral health care [21].

This study has several limitations. Among them, a primary limitation with retrospective data of childhood SES is missing data. Thus, it is possible that the results of this study are affected by selection bias, which influences the generalizability to other ethnic populations. Assessing childhood SES using retrospective CHARLS data may potentially introduce memory bias, which may potentially lead to underestimation of the association between childhood SES and edentulism. This study analyzed the SES of the parents as proxy variables, and the data of childhood SES relied on some self-reported variables. Although these variables were well validated, they may be affected by reporting bias, but other studies have suggested that the self-reported oral health variables were valid and reflected the oral health status [53, 54]. The variables in this study were available in the CHARLS data, the results were explained based on the limited variables, for instance, which may limit the association between childhood SES and edentulism. To date, the assessment of oral health in CHARLS has not been systematically validated, such as the survival bias.

Despite these limitations, this study has some strengths. For instance, this study used unique and representative mid- to late-age Chinese individuals and analyzed comprehensive variables of childhood SES. To the best of our knowledge, this study is the first study using a large sample to analyze the association between childhood SES and edentulism in mid- to late-age adults in LMICs. Regardless of the childhood SES, the study of adult oral health may overestimate the effects of other variables, such as adulthood SES in LMICs.

## Conclusion

This study provided evidence on the life course that childhood SES is related to edentulism in a representative sample of mid- to late-age Chinese individuals. This study also identified certain predictive factors related to edentulism, including education of the parents, neighbor’s help and relationships, and drinking and smoking of father, which may help policy makers and researchers to target optimal recommendations and intervention strategies to address childhood SES disparities in LMICs. Future research should focus its attention on the pathways and mediators to estimate the effects of childhood exposures on outcomes. This work highlights the measurement gap of childhood SES, and high-quality data available influence the results of the association between childhood SES and adult oral health. It is urgent to build a national representative birth cohort data in China, but so far it has not attracted the attention of the Chinese government.

## Data Availability

Please contact China Health and Retirement Longitudinal Study (CHARLS) for data requests. http://charls.pku.edu.cn/zh-CN

## Abbreviations

CHARLS: Chinese respondents in a Health and Retirement Longitudinal Study
CI: Confidence interval
CVD: Cardiovascular disease
LMICs: Low- and middle-income countries
OR: Odds ratio
SAGE: Study on global AGEing and adult health
SES: Childhood socioeconomic status
WHO: World Health Organization
